# Advances of SIRT4 in cancer metabolism and therapy

**DOI:** 10.1002/pdi3.17

**Published:** 2023-07-30

**Authors:** Xiaohan Yue, Yulu Shi, Qing Luo

**Affiliations:** ^1^ Chongqing Engineering Research Center of Stem Cell Therapy Department of Pediatrics Children’s Hospital of Chongqing Medical University National Clinical Research Center for Child Health and Disorders Ministry of Education Key Laboratory of Child Development and Disorders China International Science and Technology Cooperation Base of Child Development and Critical Disorders Chongqing China

**Keywords:** cancer, cancer metabolism, cancer therapy, SIRT4

## Abstract

The Sirtuins family consists of SIRT1‐SIRT7, which belong to class III of the histone deacetylases, a family of highly conserved NAD (nicotinamide adenine dinucleotide)‐dependent enzymes expressed in the nucleus, cytoplasm, and mitochondria. In addition to having ADP‐ribosyltransferase, NAD+‐dependent deacetylase, lipoamide, and long‐chain deacetylase activities, it can also regulate the function of substrate proteins through ADP‐ribosylation, diacylation, and long‐chain deacylation. These enzyme activities also confer many critical biological functions on SIRT4, making SIRT4 involved in many mitochondrial energy metabolic processes, such as promoting insulin secretion, participating in the glycolytic process in concert with glycolysis inhibitors, inhibiting glutamate dehydrogenase from regulating glutamine metabolism, and participating in reactions such as DNA damage. Because SIRT4 has such diverse functions, it plays a role in the metabolism and treatment of tumors. Here, we review the progress of SIRT4 research in tumor metabolism and therapy.

## 1 INTRODUCTION

**FIGURE 1:**
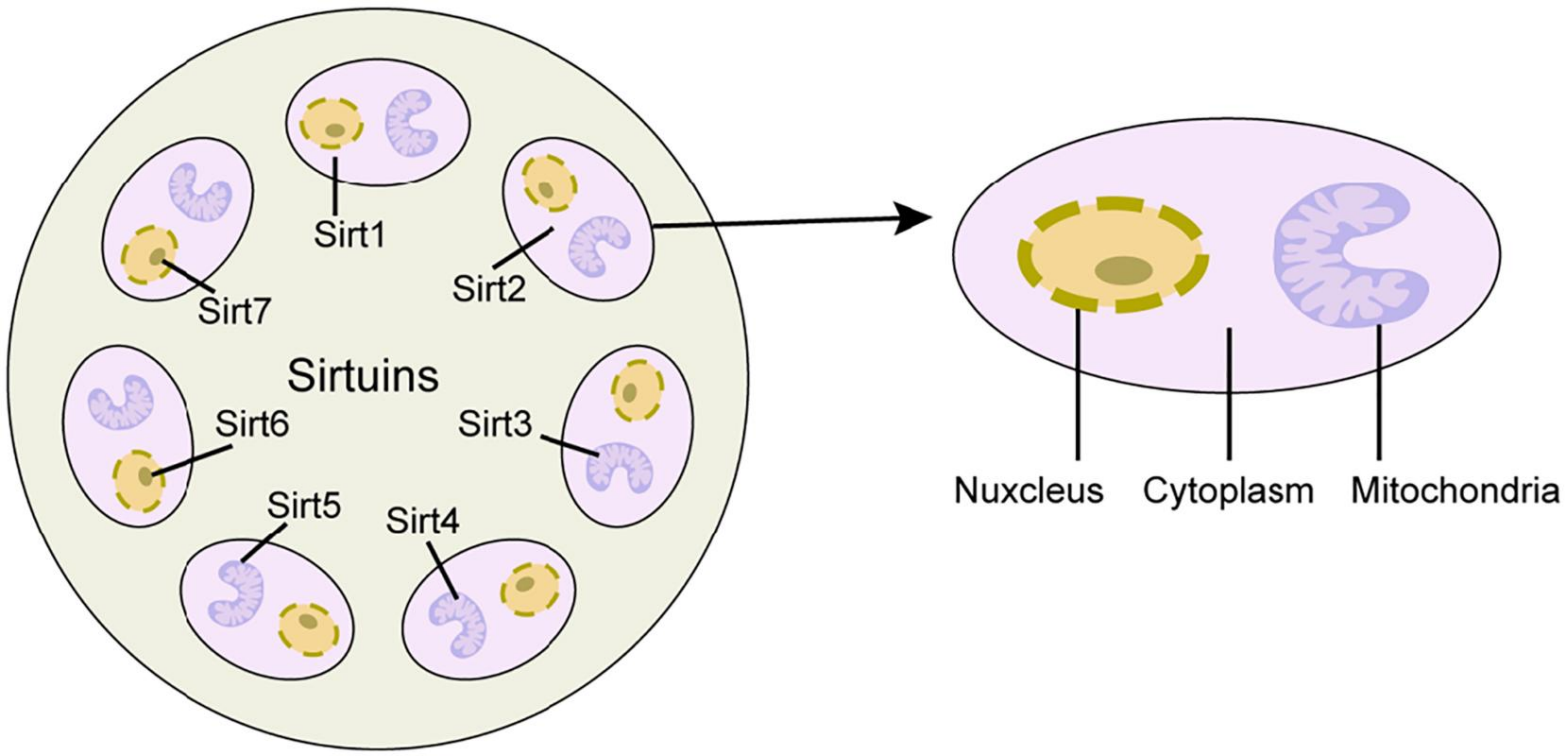
Cellular localization of the nuclear, cytoplasmic, and mitochondrial sirtuins.

Sirtuins have been studied for long‐chain deacetylation and lipoamide activity in addition to NAD‐dependent deacetylation and ADP‐ribosylation.^1^, ^2^ Sirtuins regulate cellular lifespan, control the onset of many age‐related diseases, and regulate metabolic homeostasis.^3^ SIRT4 is a member of the Sirtuins family (SIRT1‐ 7), a family of protein deacetylases and ADP‐ribosylases involved in various cellular processes, including maintenance of genomic stability and regulation of metabolism.^4^ SIRT4 is mainly found in mitochondria, and some evidence suggests that SIRT4 is a “bridge protein” between mitochondrial metabolism and tumorigenesis.^5^ SIRT1,2 located mainly in the cytoplasm, SIRT3,4 and SIRT5 are mainly in the mitochondria, and SIRT6,7 mainly in the nucleus (Figure 1). All sirtuins have conserved structural domains of deacetylases, commonly referred to as “lysine deacetylases”, which, unlike acetyltransferases, can deacetylate from lysine residues.^6^ SIRT4 is aberrantly expressed in colorectal, breast, prostate, and hepatocellular carcinomas and plays an essential role in tumor cell proliferation, differentiation, apoptosis, genomic stability, energy metabolism, DNA damage, and stress response.^7^, ^8^ Regulate adipogenesis; in addition to this, SIRT4 enhances the TORC1 signaling pathway by inhibiting the ability to convert glutamine to α‐ketoglutarate in a passive manner; thus, we see that SIRT4 can integrate nutrient input with retrograde mitochondrial signaling to maintain the balance between anabolic and catabolic pathways.^9^

SIRT4 has adenosine diphosphate ribosyltransferase (PARP) activity, which plays an essential role in DNA damage repair and apoptosis; SIRT4 deacetylates, and deacetylation causes DNA to wrap more tightly around the histone core, making transcription factors less likely to bind to DNA, resulting in lower levels of gene expression. SIRT4 does not exhibit NAD‐dependent deacetylase activity but instead uses NAD to activate ADP‐nucleotide glutamate dehydrogenase (GDH),^10^ thereby inhibiting GDH to promote DNA damage repair.^4^ SIRT4 has lysine deacetylase activity,^11^ while pyruvate dehydrogenase (PDHC) depends on lysine lipid acylation.^12^ PDHC is the key enzyme that catalyzes the conversion of pyruvate to acetyl CoA^13^, ^14^; thus, SIRT4 can affect glucose metabolism by inhibiting PDHC. SIRT4 can control fatty acid oxidation by inhibiting the transcriptional activation of PPARA. The stability of cellular metabolism plays a crucial role in maintaining the health of the body, and an essential hallmark of cancer is the reconstitution of metabolism. More and more studies confirm the inextricable relationship between cancer and metabolism. SIRT4 responds to changes in cellular nutrient availability by controlling energy metabolism, and in this way, SIRT4 plays an irreplaceable role in glucose metabolism, amino acid metabolism, and tumor therapy.

## 2 LOCATION, STRUCTURE, AND FUNCTION OF SIRT4

**FIGURE 2:**
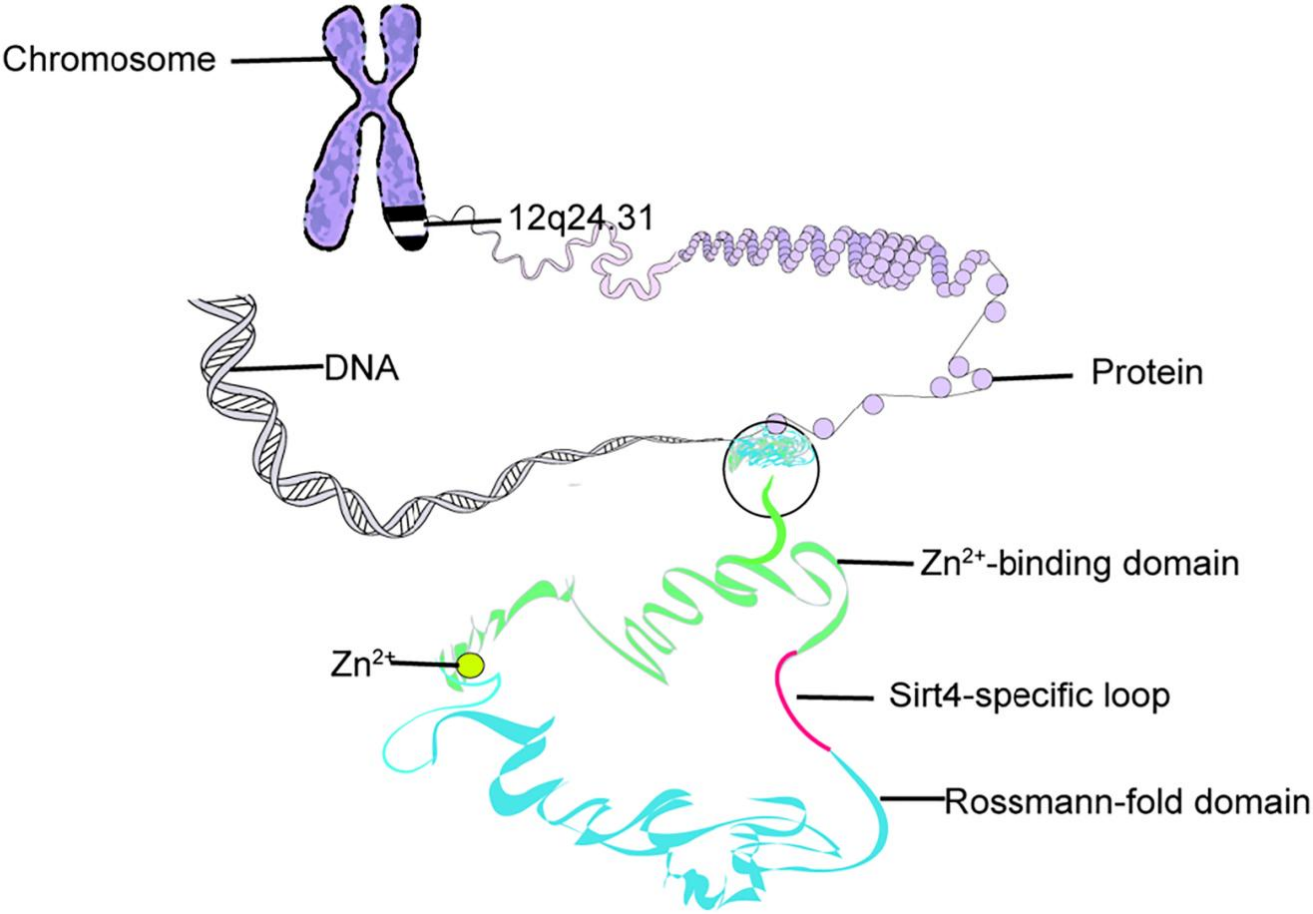
Structure of SIRT4, including chromosome location, the Zn2+‐binding domains, Sirt4‐specific loop, and Rossmann‐fold domain.

SIRT4 is highly expressed in the heart, kidney, liver, and brain and is located mainly in the mitochondrial matrix, with a small amount in the cytoplasm and nucleus. SIRT4 is located on chromosome 12q24.23‐q24.31^15^ and expressed in islets of Langerhans and aggregates with insulin‐expressing β‐cells.^11^, ^16^, ^17^ Sirtuins include many isoforms. Furthermore, SIRT4 is distinguished from other subtypes by its unique structure. After constructing a model of SIRT4, it was found that SIRT4 has a zinc‐binding domain and a Rossmann folding domain with active sites and nucleotides between them. At the same time, the catalytic activity of Sirt4 is mainly derived from two structures, one of which researchers named “Sirt4‐loop”, which is located deep in the catalytic core and contributes to substrate binding while limiting the associated active site kinetics; the other structure is a channel with a catalytic core branching off from the acyllysine binding channel^15^ (Figure 2).

**FIGURE 3:**
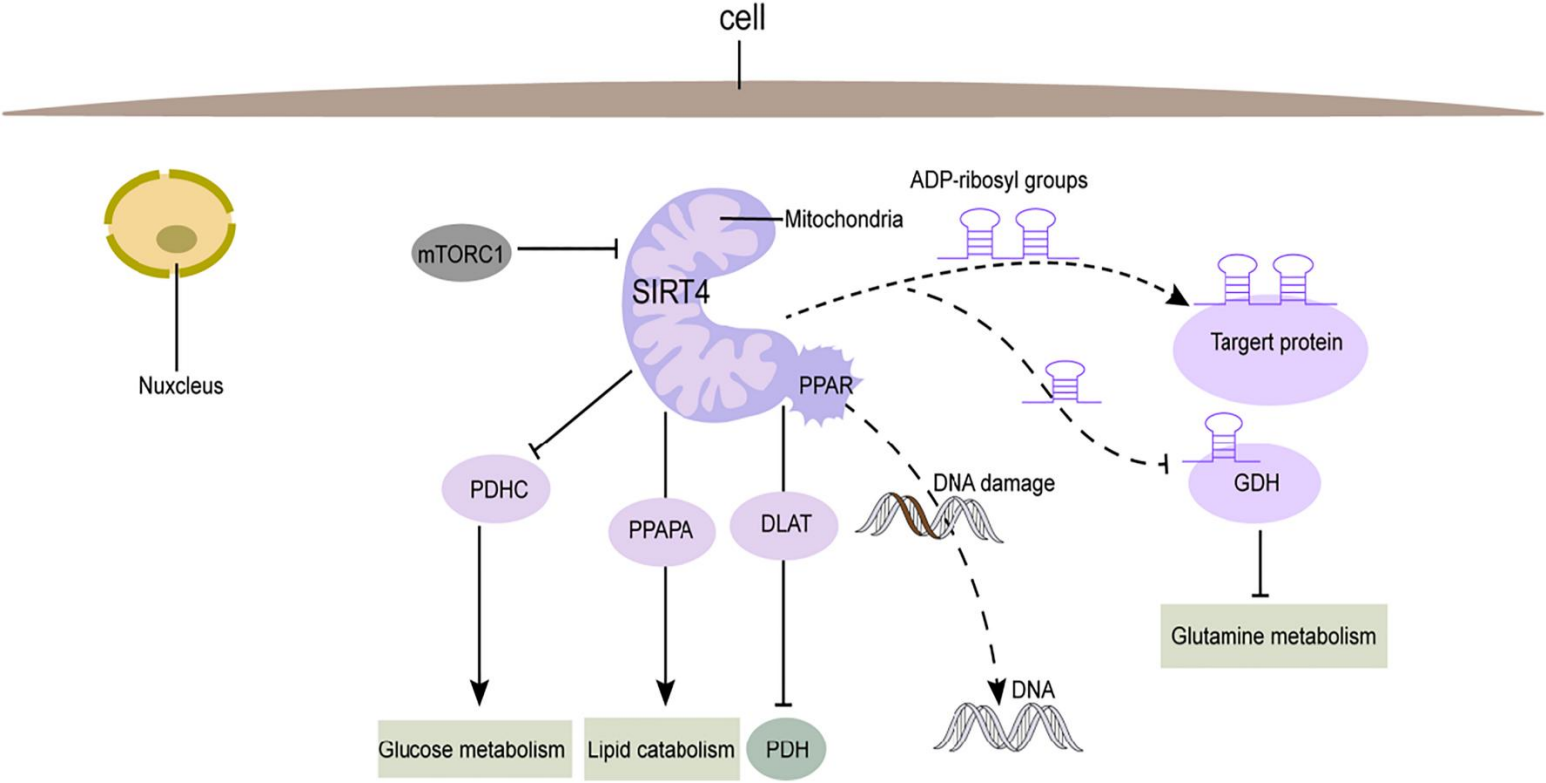
Function of SIRT4. mTORC1 regulates SIRT4 expression and glutamine metabolism. SIRT4 inhibits PDHC to regulate glucose metabolism. SIRT4 inhibits the transcriptional activation of PPARA to control fatty acid oxidation. GDH is ribosylated by ADP and inhibited by SIRT4. SIRT4 catalyzes ADP ribosyl transfer.

SIRT4 has a potent enzymatic activity that is not yet fully understood, and it is now known that SIRT4 is associated with many cellular metabolic processes, such as insulin secretion, amino acid metabolism, and lipid metabolism. SIRT4 has adenosine diphosphate ribosyltransferase (PARP) activity, which plays a vital role in DNA damage repair and apoptosis; SIRT4 deacetylation allows DNA to be wrapped more tightly around the histone core, making transcription factors less likely to bind to DNA and thus reducing gene expression levels. PDHC is the key enzyme that catalyzes the conversion of pyruvate to acetyl CoA.^13^, ^14^ SIRT4 has lysine deacetylase activity,^15^ and pyruvate dehydrogenase (PDHC) depends on lysine lipid acylation.^12^, and thus SIRT4 can influence glucose metabolism by inhibiting PDHC, which can affect sugar metabolism. SIRT4 can control fatty acid oxygenation by inhibiting the transcriptional activation of PPARA. Mitochondria are energy‐generating structures in cells that produce ROS during oxidative phosphorylation, and prolonged oxidative stress can damage mitochondria. At the same time, SIRT4 promotes apoptosis by promoting the accumulation of ROS while inhibiting HO‐1 upregulation, which is vital for regulating the tumor microenvironment^18^ (Figure 3).

## 3 SIRT4 AND CANCER

SIRT4 is aberrantly expressed in many tumors, and clarification of the expression of SIRT4 in individual tumors, as well as the relationship between SIRT4 and various tumorigenesis and progression, is essential to further investigating new therapeutic modalities for tumors. In a study examining SIRT4 in 168 groups of laryngeal squamous cell carcinoma tissues and normal paraneoplastic tissues, SIRT4 was expressed lower in laryngeal squamous cell carcinoma tissues compared to normal paraneoplastic tissues. It was significantly associated with histological grade, T‐classification, clinical stage, lymph node metastasis, and recurrence in laryngeal squamous cell carcinoma patients, while in vitro experiments showed that knockdown of SIRT4 promoted LSCC cell proliferation and migration. At the same time, SIRT4 overexpression inhibited the proliferation and migration of laryngeal squamous cell carcinoma cells.^4^ SIRT4 was shown to be expressed predominantly in glia cells and ADP‐ribosylate GDH. In a study related to gliomas, SIRT4 was shown to be lowly expressed in gliomas and to be less expressed with increasing tumor cell malignancy, survival analysis showed that patients with high SIRT4 expression had higher survival rates than those with low expression,^19^ and overexpression of SIRT4 had a protective effect on glial cell excitability.^20^ SIRT4 is downregulated in breast cancer using the TCGA breast cancer database analysis, and SIRT4‐deficient mice are more likely to exhibit tumor formation and lung metastasis.^21^ In a recent prostate cancer study, overexpression of SIRT4 inhibited the proliferation, migration, and invasive ability of prostate cancer cells and promoted apoptosis of prostate cancer cells. In contrast, further studies indicated that SIRT4 could inhibit the proliferation, migration, and invasive ability of prostate cancer cells by inhibiting glutamine metabolism.^22^ Current studies on SIRT4 in tumors prefer SIRT4 as a tumor suppressor, SIRT4 is a metabolism‐related factor with multiple enzymatic activities, and SIRT4 can inevitably have some effect on the malignant biological behavior of tumor cells by regulating the metabolism of tumor cells (Figure 3).

## 4 SIRT4 AND CANCER METABOLISM

### 4.1 SIRT4 and glucose metabolism

Nutrients are essential for the average growth of tumor cells, among which the metabolism of glucose and amino acids is more studied, and SIRT4 also plays a role in the metabolism of glucose and amino acids, so SIRT4 can also have an impact on the malignant biological behavior of tumor cells by regulating their metabolism. SIRT4 is expressed in pancreatic islets and localized to insulin‐expressing β‐cells. A deficiency of SIRT4 in insulin‐producing INS‐1E cells results in increased insulin secretion.^17^ GDH is known to promote the metabolism of glutamate and glutamine and the production of ATP, which promotes insulin secretion. Deletion of SIRT4 in insulinoma cells activates GDH, which upregulates amino acid‐stimulated insulin secretion.^10^ GDH is ribosylated by ADP and inhibited by SIRT4, inhibiting leucine‐mediated insulin secretion.^23^, ^24^ Silencing of UHRF1 significantly inhibited aerobic glycolysis in pancreatic cancer cells, while further analysis showed that SIRT4, a downstream target of UHRF1, and inhibition of SIRT4 by UHRF1 could, in turn, promote aerobic glycolysis,^25^ which at the same time left us with the question, “whether increasing the activity of SIRT4 could inhibit aerobic glycolysis in tumor cells?” Of course, it remains to be verified by studies. The glycolytic activity affected glutamine metabolism in untransformed mammary epithelial cells and several breast cancer cells. Without cell specificity, SIRT4 expression showed a time‐dependent increase and a significant decrease in GDH activity when breast cancer cells were cultured under low glucose conditions. SIRT4 expression was upregulated after restriction of glycolysis, and SIRT4 expression was transcriptionally coblocked by the transcriptional coblocker CtBP, which promotes GDH activity, CtBP function requires an adequate supply of glucose, so is it possible that the CtBP‐SIRT4‐GDH axis may coordinate glucose and glutamine metabolism? Moreover, this conjecture was supported by subsequent studies.^26^ Moreover, glucose uptake was unaffected by SIRT4 expression in prostate cancer studies.^27^ In conclusion, the above findings suggest that SIRT4 may affect glucose metabolism in different tumors.

### 4.2 SIRT4 and amino acid metabolism

Glutamine (Gln), the most abundant amino acid in the body, is catabolized to α‐ketoglutarate by two deamination reactions, the first requiring glutaminase (GLS) to produce glutamate and the second acting through glutamate dehydrogenase (GDH) or transaminase.^17^, ^28^ GDH has been shown to be the true substrate of SIRT4, which is essential for Gln homeostasis.^10^, ^26^, ^29^ Gln catabolism is essential for energy production and cell survival in the absence of glucose. Gln catabolism occurs mainly in mitochondria and is an important source of cellular ammonia, which can induce autophagy in tumor cells.^30^, ^31^ Gln plays an important role in the growth of certain tumor cells. Previous studies have shown that Gln is an important signaling molecule.^32^

SIRT4 can suppress tumor activity by inhibiting mitochondrial GDH metabolism.^33^ GDH converts Glu to α‐ketoglutarate in mitochondria, regulated by SIRT4‐mediated ADP ribosylation.^10^, ^27^ SIRT4 catalyzes the transfer of the ADP ribosyl fraction from NAD to glutamate dehydrogenase (GDH)^10^ (Figure 3), thereby inhibiting GDH activity while restricting the metabolism of Glu and Gln to produce ATP. In 2013, Alfred Csibi et al. showed that through transcriptional repression by SIRT4, mTORC1 activates glutamate dehydrogenase (GDH), which promotes glutamine anaplerosis; in addition to this, subsequent studies indicated that in liver tumors, the mTORC1 pathway could promote Gln metabolism and cell proliferation by inhibiting SIRT4.^27^ Interestingly, some researchers pointed out that the previous findings may only apply to cells in the pathological state, whereas in the physiological state, SIRT4 positively regulates TORC1 signaling.^9^ In a glioblastoma study, it was suggested that SIRT4 might indirectly inhibit Gln production by reducing GS levels^20^; in addition to this, SIRT4 can also upregulate GLS levels in colon cancer cells,^34^ which catalyze the deamination of Gln to produce Glu and NH4+.^32^ The DNA damage response leads to cell cycle arrest,^35^ and DNA damage response defects usually lead to increased mutations in newly synthesized DNA, an accumulation of chromosomal instability and tumorigenesis.^33^, ^36^, ^37^ After DNA damage, Gln catabolism is temporarily halted, which inhibits the cell cycle and even brings it to a standstill, one of the known mediators of the DNA damage response in the control of GDH catabolism is SIRT4. Typically, SIRT4 senses energy metabolism after DNA damage blocks the cell cycle and inhibits tumor formation by blocking glutamine metabolism.^38^

Using Ingenuity Pathway Analysis, the scientists tested the enrichment of the putatively interacting pathways. They found that “valine, leucine, and isoleucine metabolism” is one of the top pathways containing SIRT4‐binding proteins. That knockdown of SIRT4 in mouse liver mitochondria also reduced leucine and isoleucine metabolism. Methylcrotonyl coenzyme A carboxylases A and B (MCCA and MCCB) are interacting proteins that form nondodecameric enzyme complexes in the leucine oxidation pathway.^39^ Branched‐chain amino acids are the most consumed metabolites during adipocyte differentiation. In a pancreatic ductal adenocarcinoma (PDAC) study, SIRT4 deacetylated branched‐chain amino acid transaminase 2 (BCAT2) at lysine 44 (K44), and BCAT2 was stabilized by SIRT4 deacetylation, thereby promoting catabolism of branched‐chain amino acids,^40^ whereas methylcrotonyl coenzyme A carboxylase complex (MCCC) was previously shown to interact with SIRT4 and was considered a putative substrate for SIRT4.^39^ A recent study found that SIRT4 can inhibit methionine metabolism via MAT2A methylation, inhibiting hepatocellular carcinoma progression.^41^ As mentioned above, SIRT4 not only regulates glutamine metabolism in tumors but is also involved in regulating valine, leucine, isoleucine, and methionine metabolism.

### 4.3 SIRT4 and lipid metabolism

In lipogenic cells, the activation of SREBP1c, the primary transcriptional regulator of lipid metabolism, depends mainly on TORC1 activity, and studies in hepatocytes found that the SIRT4‐TORC1 axis activates the transcriptional process of SREBP1c and increases the number of measured lipids, indicating that SIRT4 can activate the lipogenic response in hepatocytes.^9^ By examining the livers of patients with NAFLD, Tao et al. found that the expression of SIRT1, SIRT3, SIRT5, and SIRT6 was decreased in the NAFLD group compared to the control group, while the expression of SIRT4 was increased.^42^ PPARα is the primary transcriptional activator of fatty acid catabolism in the liver,^43^, ^44^ SIRT4 is shown to activate fatty acid catabolism through NAD+, and SIRT1 inhibits the expression of PPARα target genes. It was found that SIRT4 suppresses the expression of PPARα target genes by decreasing PPARα activity, thereby inhibiting hepatic fat oxidation. Malonyl coenzyme A is a critical metabolite that inhibits lipolysis metabolism and promotes lipid synthesis; in tests with mouse adipocyte cell lines, SIRT4 was found to inhibit fatty acid oxidation and promote lipid anabolism by inhibiting malonyl coenzyme A‐decarboxylase.^45^ In a departure from previous studies, a study in *Drosophila* showed that *Drosophila* knockouts of SIRT4 showed impaired triglyceride utilization during fasting, maintaining higher levels of triglycerides, particularly for lipids with chain lengths of C18 or greater molecular weight,^46^ suggesting that SIRT4 may play a different role in *Drosophila* than in mouse adipocytes. However, unfortunately, there are no studies involving SIRT4 related to lipid metabolism in tumors, and more and more relevant studies will appear in the future.

## 5 SIRT4 AND CANCER THERAPY

Among the various studies on cancer, the ultimate aim is to provide new therapeutic directions. Currently, blocking the metabolic pathways of tumor cells is being actively explored as a new cancer treatment strategy. In 2013, Jeong et al. showed that SIRT4 acts as a tumor suppressor by regulating glutamine metabolism in hepg2 hepatocellular carcinoma cells and PC3 human prostate cancer cells, and SIRT4 knockout mice spontaneously developed lung cancer^38^; SIRT4 was expressed lower than average tissue in bladder cancer, T‐cell leukemia, lung cancer, ovarian cancer, breast cancer, liver cancer, laryngeal squamous cell carcinoma, thyroid cancer, and other tumors, suggesting that SIRT4 may have a tumor suppressor role in some cancers.

### 5.1 Synergistic glutamine metabolism

Tumor‐selective targeting of glutamine metabolism has been proposed as a therapeutic strategy. Gln catabolism is essential for energy production and cell survival in the absence of glucose, and blocking glutamine metabolism has excellent potential for certain glutamine metabolism‐dependent cancers. Breast cancer showed significant antitumor effects^47^; in colorectal cancer studies, overexpression of SIRT4 was found to inhibit the metabolism of glutamine while also inhibiting the proliferation of rectal cancer cells through synergistic glucose inhibitors^48^; glutamine transporter protein inhibitor V‐9302 was able to selectively block triple‐negative breast cancer (TNBC) cells' glutamine uptake and simultaneously enhance the immune response of T cells, suggesting that preferential inhibition of glutamine metabolism in tumor cells may represent a promising targeted therapy.^49^ SIRT4 inhibits prostate cancer cell invasion and migration through inhibition of glutamine metabolism^22^; in the context of radiation therapy of tumors, Mukha et al. found that prostate cancer cells have a high glutamine requirement, and inhibition of GLS to target glutamine metabolism in prostate cells resulted in significant radiosensitization of cancer cells,^50^, ^51^ suggesting that inhibition of glutamine metabolism can be used as a prognostic biomarker and therapeutic target for radiosensitization of prostate cancer. SIRT4 inhibits B‐cell lymphoma proliferation by inhibiting mitochondrial glutamine metabolism^52^; in addition to this, SIRT4 inhibits B‐cell lymphoma proliferation by inhibiting glutamine metabolism from suppressing the malignant biological behavior of thyroid cancer cells^53^; In conclusion, these findings suggest the potential of SIRT4 for tumor‐targeted therapeutic aspects, especially its potential to synergize with glutamine metabolism inhibitors for cancer treatment.

### 5.2 Synergistic chemotherapeutic agents

In 2016, Yu et al. found that SIRT4 was significantly downregulated in colorectal cancer tissues through analysis of 89 colorectal cancer cases and that SIRT4 also increased the sensitivity of colorectal cancer cells to the chemotherapeutic drug 5‐fluorouracil through cell cycle inhibition^48^; subsequently, in a study on colorectal cancer (CRC), researchers noted that SIRT4 might enhance the sensitivity of CRC cells to 5‐FU and oxaliplatin chemosensitivity.^54^ In a study on breast cancer, SIRT4 was found to enhance the sensitivity of breast cancer cells to tamoxifen. In contrast, SIRT4 enhanced tamoxifen sensitivity in breast cancer cells by inhibiting the STAT3 signaling pathway.^55^ Cisplatin is one of the most effective chemotherapeutic agents for bladder cancer, but its resistance is a major obstacle in cancer treatment. Researchers have identified miR‐424 associated with cisplatin resistance, and it has been suggested that SIRT4 is a downstream target gene of miR‐424, and miR‐424 mediates cisplatin resistance by inhibiting SIRT4.^56^ Can this help researchers further explore the reduction of cisplatin resistance by promoting the accumulation of SIRT4? Doxorubicin (DOX) is an effective anthracycline‐based chemotherapeutic agent. However, its induced cardiotoxicity (DIC) limits its application in cancer therapy, and one study found that SIRT4 overexpression can inhibit Akt/mTOR‐dependent autophagy from preventing DIC^57^; in the antitumor aspect of herbal medicine, it was found that paeoniflorin can inhibit STAT3 activation and prevent DIC by promoting SIRT4 expression to enhance the sensitivity of estrogen receptor‐positive breast cancer cells to tamoxifen; Sorafenib is a first‐line dual‐target inhibitor that can target both serine‐threonine kinase Raf and tyrosine kinase VEGFR/PDGFR, while SIRT4 can synergize with sorafenib to make hepatocellular carcinoma cells more sensitive to sorafenib‐targeted therapy by inhibiting methionine metabolism.^41^ In conclusion, these results highlight the potential application of SIRT4 in cancer therapy.

## 6 SUMMARY

Mitochondria are critical for maintaining energy homeostasis in cellular and organismal physiology, and abnormalities in mitochondrial function are associated with a large number of studies now showing that mitochondria can be targets for tumor therapy. SIRT4 is aberrantly expressed in a variety of tumor diseases; studies in breast cancer have found that SIRT4 expression increases with time when breast cancer cells are cultured under low glycemic conditions. SIRT4 has an important role in the regulation of amino acid metabolism in tumors, while the mechanisms of SIRT4's regulation of glucose metabolism and regulation of lipid metabolism remain to be further discovered. In tumor therapy, SIRT4 overexpression was found to inhibit glutamine metabolism and also inhibit the proliferation of rectal cancer cells by synergizing with glucose inhibitors; SIRT4 inhibits the invasion and migration ability of prostate cancer cells by inhibiting glutamine metabolism; besides SIRT4's ability to synergize with glutamine metabolism for tumor treatment, SIRT4 can also synergize with antitumor drugs. For example, SIRT4 can enhance the drug sensitivity of colorectal cancer cells to 5‐FU and oxaliplatin; SIRT4 can enhance the sensitivity of breast cancer cells to tamoxifen, etc. In conclusion, whether it is the regulation of energy metabolism in tumor cells by SIRT4 or exerting certain therapeutic effects on tumors through synergistic glutamine metabolism and synergistic antitumor drugs, it indicates that the effects of SIRT4 on tumor cells have certain research prospects.

## AUTHOR CONTRIBUTIONS

Xiaohan Yue conceived and designed the content of this review and is responsible for the visual presentation. Xiaohan Yue wrote the manuscript with the help of Qing Luo and Yulu Shi. Xiaohan Yue contributed to the final version of manuscript. ALL authors contributed to the artical and approved the submitted version.

## CONFLICT OF INTEREST STATEMENT

The authors declare no conflict of interest.

## ETHICS STATEMENT

Not applicable.

## Data Availability

Data sharing not applicable to this article as no datasets were generated or analyzed during the current study.
